# Inositol pyrophosphate kinases in health and disease

**DOI:** 10.1002/1873-3468.70192

**Published:** 2025-10-23

**Authors:** Changchang Xing, Chao Wang, Yuanyuan Chen, Weiwei Cheng, Zhaolei Jiang, Alex F. Chen, Chenglai Fu

**Affiliations:** ^1^ Department of Cardiothoracic Surgery, Xinhua Hospital Shanghai Jiao Tong University School of Medicine China; ^2^ Institute for Developmental and Regenerative Cardiovascular Medicine, Xinhua Hospital Shanghai Jiao Tong University School of Medicine China; ^3^ Department of Hypertension Peking University People's Hospital Beijing China; ^4^ Department of Nuclear Medicine Xinhua Hospital Affiliated to Shanghai Jiao Tong University School of Medicine China

**Keywords:** 1PP‐InsP_5_, 5PP‐InsP_5_, InsP_8_, IP6K, PPIP5K

## Abstract

Inositol phosphates (InsPs) are intracellular signaling molecules that are essential for life. Inositol pyrophosphates, a subset of inositol phosphates, are the end products of inositol phosphate metabolism. In mammalian cells, up to 90% of inositol pyrophosphates are 5‐diphosphoinositol 1,2,3,4,6‐pentakisphosphate (5PP‐InsP_5_), which is generated by inositol hexakisphosphate kinases (IP6Ks). 5PP‐InsP_5_ can be further phosphorylated by diphosphoinositol pentakisphosphate kinases (PPIP5Ks) to generate 1,5‐bisdiphosphoinositol 2,3,4,6‐tetrakisphosphate (InsP_8_). Unlike freely diffusible molecules, 5PP‐InsP_5_ and InsP_8_ act locally at the sites where they are synthesized. Thus, individual IP6K and PPIP5K enzymes perform specific functions. Preclinical and clinical studies suggest that these molecules contribute to early life development, but mediate age‐related diseases beyond adulthood. In this review, we summarize the functions and mechanisms of action of every individual IP6K and PPIP5K in both physiological processes and diseases and discuss the potential applications of these inositol pyrophosphate kinases as druggable targets for disease treatment.

## Graphical abstract

In mammalian cells, IP6Ks and PPIP5Ks are the key kinases synthesizing the inositol pyrophosphates 5PP‐InsP_5_ and InsP_8_. They are essential for early development but also mediate age‐related diseases, including metabolic disorders, cardiovascular diseases, neurodegenerative diseases, and cancers. This review explores their functions and mechanisms in physiological processes and disease states, and their potential as therapeutic targets for disease intervention.

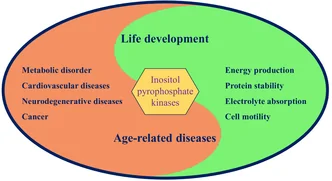

## Abbreviations

**1PP‐InsP**_**5**_, 1‐diphospho‐inositol 2,3,4,5,6‐pentakisphosphate

**5PP‐Ins(1,3,4,6)P**_**4**_, 5‐diphosphoinositol (1,3,4,6)‐tetrakisphosphate

**5PP‐InsP**_**5**_, 5‐diphosphoinositol 1,2,3,4,6‐pentakisphosphate

**Ins(1,3,4,5)P**_**4**_, inositol 1,3,4,5‐tetrakisphosphate

**Ins(1,3,4,5,6)P**_**5**_, inositol 1,3,4,5,6‐pentakisphosphate

**Ins(1,4,5)P**_**3**_, inositol 1,4,5‐trisphosphate

**InsP**_**6**_, inositol‐1,2,3,4,5,6‐hexakisphosphate

**InsP**_**8**_, 1,5‐bisdiphosphoinositol 2,3,4,6‐tetrakisphosphate

**InsPs**, inositol phosphates

**IP3K**, inositol trisphosphate 3‐kinase

**IP6K**, inositol hexakisphosphate kinase

**IPMK**, inositol polyphosphate multikinase

**IPPK**, inositol pentakisphosphate 2‐kinase

**JMJD2C**, Jumonji domain containing 2C

**KO**, knockout

**PPIP5K**, diphosphoinositol pentakisphosphate kinase

**WT**, wild‐type

**XPR1**, xenotropic and polytropic retrovirus receptor 1

**Fig. 1:**
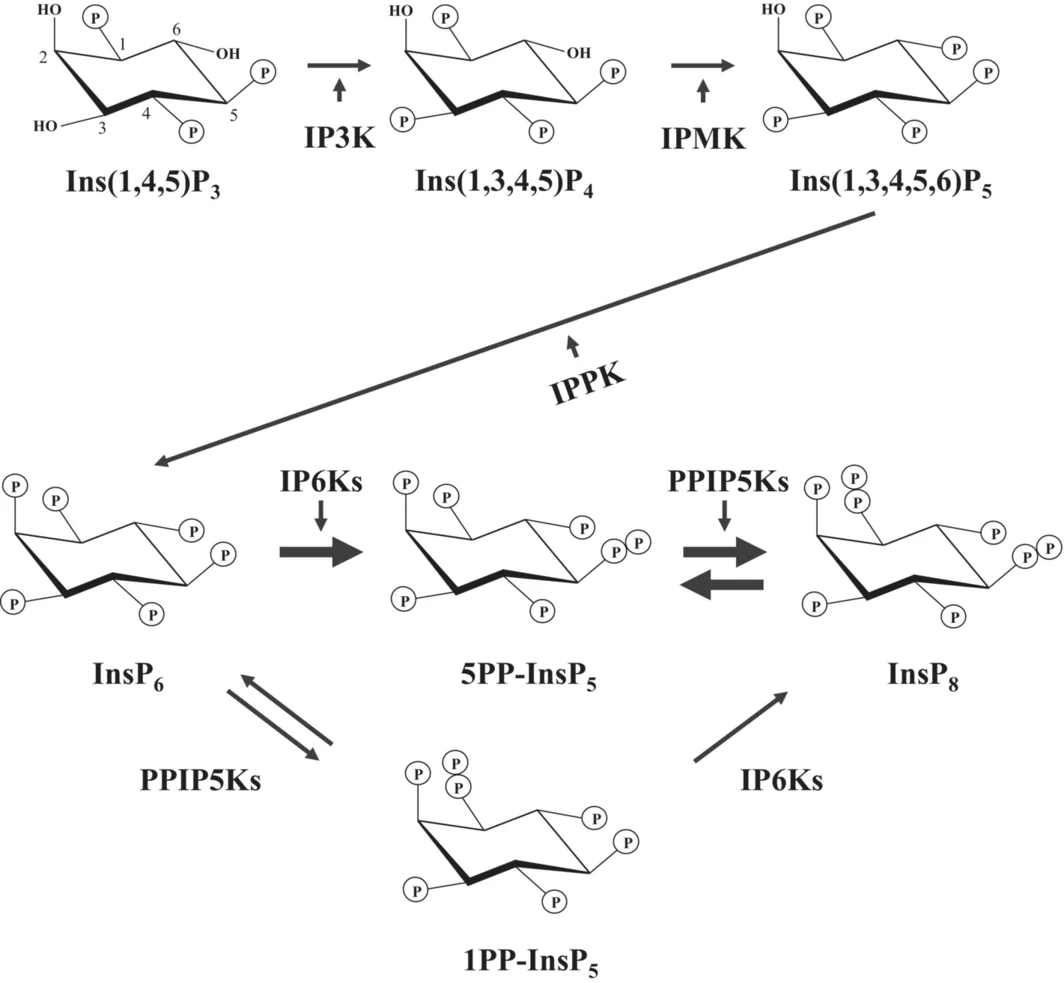
Metabolic pathway of inositol phosphates and inositol pyrophosphates. Ins(1,4,5)P_3_ can be phosphorylated by IP3K to form Ins(1,3,4,5)P_4_, which is subsequently phosphorylated by IPMK to synthesize Ins(1,3,4,5,6)P_5_. Ins(1,3,4,5,6)P_5_ is further phosphorylated by IPPK to generate InsP_6_. InsP_6_ is mainly phosphorylated by IP6Ks to form 5PP‐InsP_5_, which is phosphorylated by PPIP5Ks to generate InsP_8_. A minor portion of InsP_6_ is phosphorylated by PPIP5Ks to form 1PP‐InsP_5_, which is phosphorylated by IP6Ks to generate InsP_8_. InsP_8_ and 1PP‐InsP_5_ can be dephosphorylated by PPIP5Ks back to 5PP‐InsP_5_ and InsP_6_, respectively.

Inositol pyrophosphates belong to the family of inositol phosphates, of which the best characterized is inositol 1,4,5‐trisphosphate (Ins(1,4,5)P_3_). Acting as a ‘second messenger’, Ins(1,4,5)P_3_ triggers the release of intracellular Ca^2+^ from the endoplasmic reticulum. Ins(1,4,5)P_3_ is phosphorylated to form higher inositol phosphates and inositol pyrophosphates, which participate in multiple cellular processes. In mammalian cells, inositol trisphosphate 3‐kinase (IP3K) converts Ins(1,4,5)P_3_ to inositol 1,3,4,5‐tetrakisphosphate (Ins(1,3,4,5)P_4_). Inositol polyphosphate multikinase (IPMK) utilizes Ins(1,3,4,5)P_4_ as a substrate to synthesize inositol 1,3,4,5,6‐pentakisphosphate (Ins(1,3,4,5,6)P_5_). Ins(1,3,4,5,6)P_5_ is then phosphorylated by inositol pentakisphosphate 2‐kinase (IPPK) to generate inositol‐1,2,3,4,5,6‐hexakisphosphate (InsP_6_) [1] (Fig. 1). Both IPMK and IPPK are essential for life; null mutations of either enzyme in mice result in embryonic lethality [2, 3].

IP6Ks catalyze the phosphorylation of InsP_6_ at the fifth position of the inositol ring to produce 5PP‐InsP_5_, which is the most abundant and well‐characterized inositol pyrophosphate in mammalian cells [4]. 5PP‐InsP_5_ is a highly metabolically active molecule. In pancreatoma cells, 50% of the InsP_6_ pool is converted to 5PP‐InsP_5_ within 1 h [5]. In primary cultured hepatocytes, its cellular pool typically turns over at least 10 times every 40 min [6]. IP6Ks can also phosphorylate Ins(1,3,4,5,6)P_5_ to generate 5PP‐Ins(1,3,4,6)P_4_ [7, 8]. However, the cellular content of 5PP‐Ins(1,3,4,6)P_4_ is extremely low, and its functions in mammalian cells remain a mystery. Mammalian cells express three IP6Ks. Deletion of an individual Ip6k in mice does not result in embryonic lethality but leads to developmental defects [9, 10, 11, 12]. Although IP6Ks are critical for early life development, they also contribute to metabolic disorders, cardiovascular diseases, and neuronal degenerative diseases [4]. This indicates that IP6Ks contribute to body development until young adulthood, but beyond this stage, they mediate the development of age‐related diseases.

PPIP5Ks (PPIP5K1 and PPIP5K2) are capable of phosphorylating InsP_6_ to generate 1‐diphospho‐inositol 2,3,4,5,6‐pentakisphosphate (1PP‐InsP_5_), but they predominantly phosphorylate 5PP‐InsP_5_ *in vivo* to synthesize InsP_8_. The combined amount of 5PP‐InsP_5_ and InsP_8_ surpasses 95% of total inositol pyrophosphates, representing the major metabolic pathway of inositol pyrophosphates in mammalian cells (Fig. 1). 5PP‐InsP_5_ and InsP_8_ exert structural modification effects by binding to their target proteins [13]. Additionally, they can induce protein pyrophosphorylation [14]. 5PP‐InsP_5_ and InsP_8_ do not freely diffuse within the cell; instead, they are produced in compartmentalized subcellular regions where IP6Ks and PPIP5Ks are localized. Thus, the functions of 5PP‐InsP_5_ and InsP_8_ are determined by the specific IP6K and PPIP5K isoforms present in these subcellular compartments [1, 4, 15].

Recently, with the advancement of various detection techniques capable of measuring mass levels of inositol pyrophosphates in cells from different species, novel inositol pyrophosphate isomers, including 2PP‐InsP_5_, 3PP‐InsP_5_, and 4/6PP‐InsP_5_, have been identified in mammalian and plant cells [16]. However, the enzymes responsible for the synthesis of these minor inositol pyrophosphates are not yet identified.

IP6Ks and PPIP5Ks appear to be the most important inositol pyrophosphate kinases in mammalian cells, and their functions have been revealed by genetic and pharmacological studies in preclinical models [17, 18, 19, 20, 21, 22, 23, 24, 25, 26, 27, 28]. The importance of IP6Ks and PPIP5Ks in humans is also demonstrated by several clinical studies [29, 30, 31, 32, 33, 34]. Here, we review the functions and mechanisms of action of each individual IP6K and PPIP5K in physiology and disease.

## Physiological functions and mechanisms of action of IP6Ks

Mammalian cells express a family of three IP6Ks, namely IP6K1, IP6K2, and IP6K3. IP6K1 and IP6K2 are expressed in almost all types of cells, whereas IP6K3 expression is tissue‐specific. Although IP6Ks share identical enzymatic activities, they perform nonredundant functions. This is because these enzymes are distributed in different subcellular areas and perform both kinase‐dependent and kinase‐independent tasks [4]. Specifically, IP6K1 is primarily located in the cytosol and at the plasma membrane, although it is also present in the nucleolus [9, 14]. IP6K2 is mainly a nuclear protein, while IP6K3 is found in the cytosol and at the cell membrane [9]. The physiological functions of each individual IP6K have been demonstrated through their respective knockout mouse models.

### Physiological functions and mechanisms of action of IP6K1

**Fig. 2:**
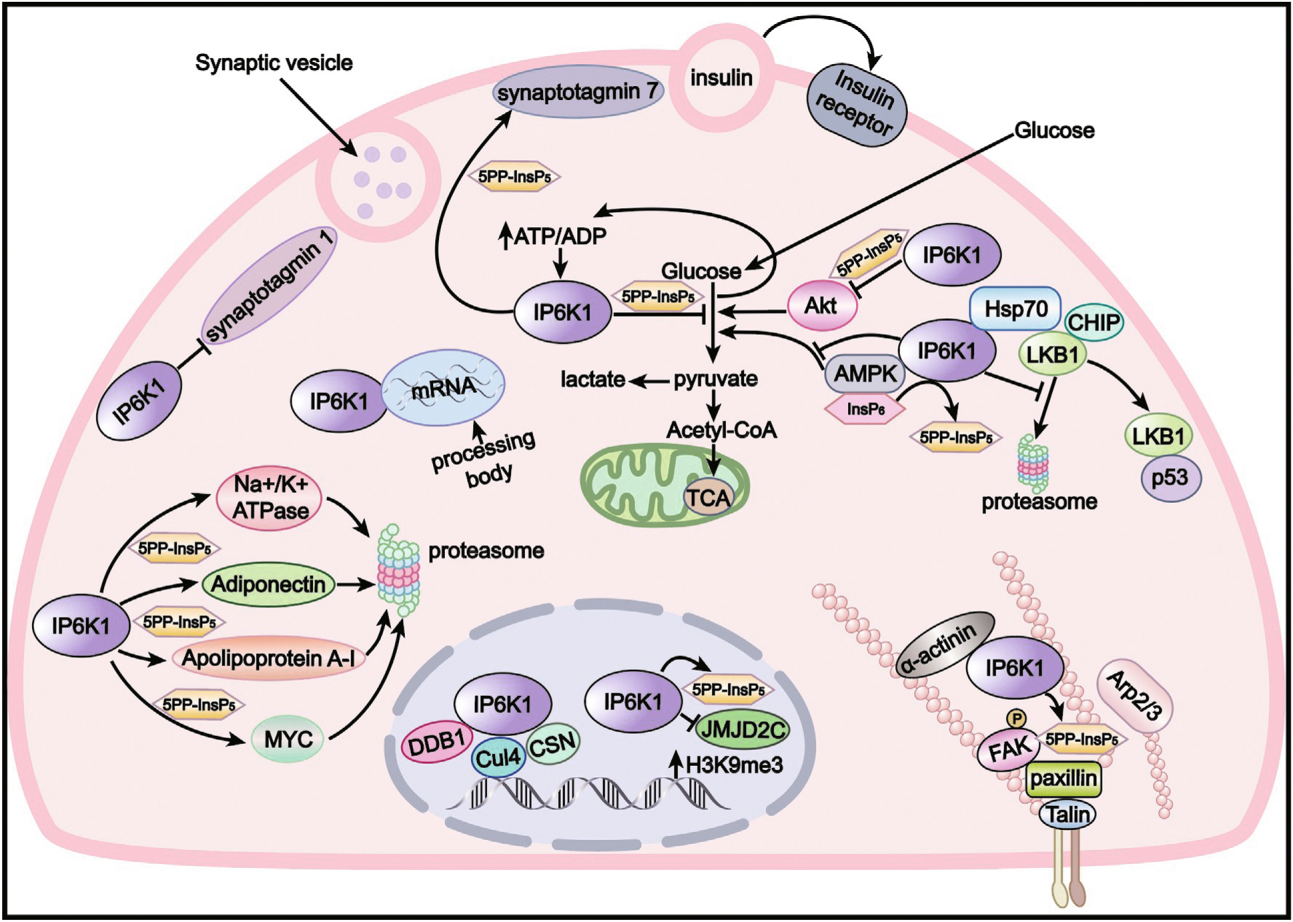
Mechanisms of action of IP6K1. In the nucleus, IP6K1 facilitates homologous recombination repair and inhibits JMJD2C‐mediated histone demethylation. In the cytosol, IP6K1 promotes the degradation of several proteins, including Na^+^/K^+^‐ATPase, adiponectin, apolipoprotein A‐I, and MYC, *via* its product 5PP‐InsP_5_. Meanwhile, it stabilizes LKB1 in a kinase‐independent manner. Additionally, IP6K1 promotes the formation of processing bodies and inhibits glycolysis by suppressing the Akt and AMPK signaling pathways. At the cell membrane, IP6K1 inhibits synaptotagmin 1‐mediated synaptic vesicle release but enhances synaptotagmin 7‐mediated insulin release. Furthermore, IP6K1 binds to focal adhesion proteins to promote cytoskeletal remodeling and focal adhesion turnover.

IP6K1 is the major isoform, accounting for up to 70% of cellular 5PP‐InsP_5_ production. Its activity can be regulated by phosphorylation. Specifically, PKD phosphorylates IP6K1 at serine 118, while PKC phosphorylates it at serine 121. Notably, double phosphorylation at these sites has been shown to enhance its catalytic activity [28]. IP6K1 is the best‐characterized isoform, with multiple cellular functions and mechanisms of action demonstrated in both *in vitro* and *in vivo* models (Table 1 and Fig. 2).

**Table 1:** The physiological functions of IP6K1.

	Mechanisms of action
Cellular functions	
1. IP6K1 inhibits Akt signaling	5PP‐InsP_5_, product of IP6K1, competes with PtdIns(3,4,5)P_3_ to bind to the PH domain of Akt
2. IP6K1 facilitates cytoskeleton remodeling and focal adhesion turnover	IP6K1 binds to α‐actinin and *via* its product 5PP‐InsP_5_ promotes FAK phosphorylation. IP6K1 also binds to the Arp2/3 complex and recruits coronin through 5PP‐InsP_5_
3. IP6K1 promotes insulin secretion	IP6K1 *via* its product 5PP‐InsP_5_ promotes synaptotagmin‐7‐mediated insulin release
4. IP6K1 promotes DNA homologous repair	IP6K1 orchestrates assembly/disassembly of the CRL4‐signalosome complex
5. IP6K1 inhibits energy consumption	IP6K1 inhibits glycolysis and mitochondrial oxidative phosphorylation. IP6K1 binds perilipin‐1 non‐catalytically to block hormone‐sensitive lipase access to lipid droplets
6. IP6K1 facilitates intracellular vesicle transport	IP6K1 promotes the binding of p150 (Glued) to dynein through its product 5PP‐InsP_5_
7. IP6K1 regulates protein expression	IP6K1 inhibits histone demethylase JMJD2C, and promotes the formation of processing bodies
8. IP6K1 regulates stability of its target proteins	IP6K1 *via* its product 5PP‐InsP_5_ promotes degradation of Na^+^/K^+^‐ATPase, MYC, adiponectin, and apolipoprotein A1. IP6K1 can also stabilize LKB1 in a kinase‐independent manner
*In vivo* functions	
1. IP6K1 promotes spermatogenesis	IP6K1 is required for proper Sertoli‐germ cell interactions and spermatid development
2. IP6K1 promotes nervous system development	IP6K1 promotes neuronal cell migration by activating the FAK signaling pathway
3. IP6K1 regulates neuronal excitability and memory	IP6K1 regulates action potential frequency and the afterhyperpolarization phase by affecting Na^+^/K^+^‐ATPase protein levels. It also regulates synaptic vesicle release and recycling
4. IP6K1 regulates blood glucose levels	IP6K1 promotes insulin secretion in pancreatic β‐cells. However, it inhibits insulin sensitivity by blocking Akt signaling
5. IP6K1 regulates hemostasis	IP6K1 promotes platelet coagulation
6. IP6K1 regulates innate immune functions	IP6K1 promotes neutrophil–platelet aggregate formation, but blocks phagocytic and bactericidal activity in neutrophils
7. IP6K1 regulates metabolite reabsorption in renal tubules	IP6K1 reduces the protein levels of Na^+^/K^+^‐ATPase, but boosts the protein levels of Na^+^/Pi cotransporters in renal tubules

### The cellular functions of IP6K1

IP6K1 is an important regulator of cell metabolism. It inhibits ATP production by altering glycolysis and mitochondrial oxidative phosphorylation [35]. Additionally, IP6K1 inhibits lipolysis through its interaction with the lipolytic regulator protein perilipin1 [36].

IP6K1 is also required for homologous recombination‐mediated DNA repair [37]. It interacts with the DDB1–Cul4A–Roc1 E3 ligase complex and mediates assembly and disassembly of the CRL4‐signalosome complex to regulate DNA repair and cell death [38].

Furthermore, IP6K1 regulates cytoskeleton remodeling and focal adhesion turnover *via* its product, 5PP‐InsP_5_ [10, 39]. IP6K1 also promotes cytoplasmic dynein‐driven vesicle transport [40].

IP6K1 can regulate protein expression levels at both the transcriptional and posttranscriptional levels. At the transcriptional level, IP6K1 interacts with Jumonji domain containing 2C (JMJD2C), a histone lysine demethylase. Through its product, 5PP‐InsP_5_, IP6K1 induces the dissociation of JMJD2C from chromatin, thereby increasing H3K9me3 levels. This leads to changes in the transcription of JMJD2C‐target genes [41]. Additionally, IP6K1 can act independently of its catalytic activity to upregulate the formation of processing bodies, which are cytoplasmic ribonucleoprotein granules that store translationally repressed mRNA [42]. At the posttranscriptional level, IP6K1, *via* its product 5PP‐InsP_5_, induces the ubiquitination and degradation of several proteins, including Na^+^/K^+^‐ATPase, MYC, adiponectin, and apolipoprotein A1 [18, 19, 21, 43]. In contrast, IP6K1 can prevent HSP70/CHIP‐dependent LKB1 degradation in a kinase‐independent manner [17].

### The *in vivo* functions of IP6K1

The *in vivo* physiological functions of IP6K1 have been characterized using *Ip6k1* knockout (KO) mice. Whole‐body deletion of IP6K1 causes deficits in multiple systems, including the male reproductive system [22, 44], the nervous system [10, 20, 45], the endocrine system [28, 46], the blood system [47], and the urinary system [21, 48]. Additionally, *Ip6k1* KO animals are smaller than their wild‐type (WT) littermates [49].

One obvious defect in *Ip6k1* KO mice is male infertility, whereas *Ip6k1* KO females are capable of becoming pregnant and bearing offspring. IP6K1 is essential for chromatoid body formation in mouse spermatids [25]. The blood‐testis barrier, as well as the interactions between germ cells and Sertoli cells, cannot form properly without IP6K1 [22, 44]. Thus, deletion of IP6K1 elicits several aberrations in male germ cell development [22].

Deleting IP6K1 leads to brain malformations, which can result in premature death [10]. IP6K1 binds to α‐actinin, a focal adhesion protein. Within focal adhesions, IP6K1 generates 5PP‐InsP_5_ to promote focal adhesion kinase phosphorylation, a key step for cell migration. Neuronal cells lacking IP6K1 display delayed migration during the embryonic stage [10]. IP6K1 also determines neuronal excitability and neurotransmitter release. Deleting IP6K1 stabilizes Na^+^/K^+^‐ATPase, leading to a lower frequency of action potentials and a specific deepening of the afterhyperpolarization phase [20]. Deletion of IP6K1 also impairs the coordination of synaptic vesicle exocytosis and endocytosis [45, 50]. IP6K1 and its product 5PP‐InsP_5_ are required for inhibiting synaptotagmin 1‐dependent synaptic vesicle fusion [51]. *Ip6k1* KO mice display impaired short‐term memory formation [52] and behavioral alterations [53].

IP6K1 functions as a metabolic sensor in pancreatic β‐cells. Glucose stimulation increases the ATP/ADP ratio, which leads to an early rise in 5PP‐InsP_5_ concentration in β‐cells [54]. Additionally, vagal stimulation and activation of muscarinic acetylcholine receptors can also activate IP6K1, leading to increased 5PP‐InsP_5_ synthesis. 5PP‐InsP_5_ promotes synaptotagmin‐7‐dependent insulin release [28]. Pancreatic β‐cells maintain a high basal concentration of 5PP‐InsP_5_. Deleting IP6K1 impairs insulin exocytosis [46]. Paradoxically, *Ip6k1* KO mice demonstrate decreased plasma insulin levels but increased insulin sensitivity. This is because 5PP‐InsP_5_ is an endogenous inhibitor of Akt. Deletion of IP6K1 removes 5PP‐InsP_5_‐dependent Akt inhibition, leading to increased insulin sensitivity [55].

In the blood system, IP6K1 maintains hemostasis by regulating platelet aggregation. Deleting IP6K1 lowers polyphosphate levels in platelets, resulting in impaired coagulation. *Ip6k1* KO mice demonstrate longer tail bleeding time and resistance to thromboembolism [47]. In neutrophils, IP6K1, *via* its product 5PP‐InsP_5_, blocks Akt activity, thereby inhibiting neutrophil phagocytic and bactericidal abilities [56]. Additionally, IP6K1 is required for neutrophil–platelet aggregation [57].

In the urinary system, IP6K1 affects Na^+^ and water reabsorption in the kidney by regulating Na^+^/K^+^‐ATPase protein levels. IP6K1 generates 5PP‐InsP_5_ to drive Na^+^/K^+^‐ATPase endocytosis and degradation. Deleting IP6K1 upregulates Na^+^/K^+^‐ATPase, leading to increased fluid retention [21]. Additionally, IP6K1, together with IP6K2, regulates the protein levels of the Na^+^/Pi cotransporters NaPi‐IIa and NaPi‐IIc. Depletion of IP6K1 and IP6K2 from renal tubules leads to general tubular dysfunction, including albuminuria, increased diuresis, and reduced urinary osmolarity [48].

### Physiological functions and mechanisms of action of IP6K2

**Fig. 3:**
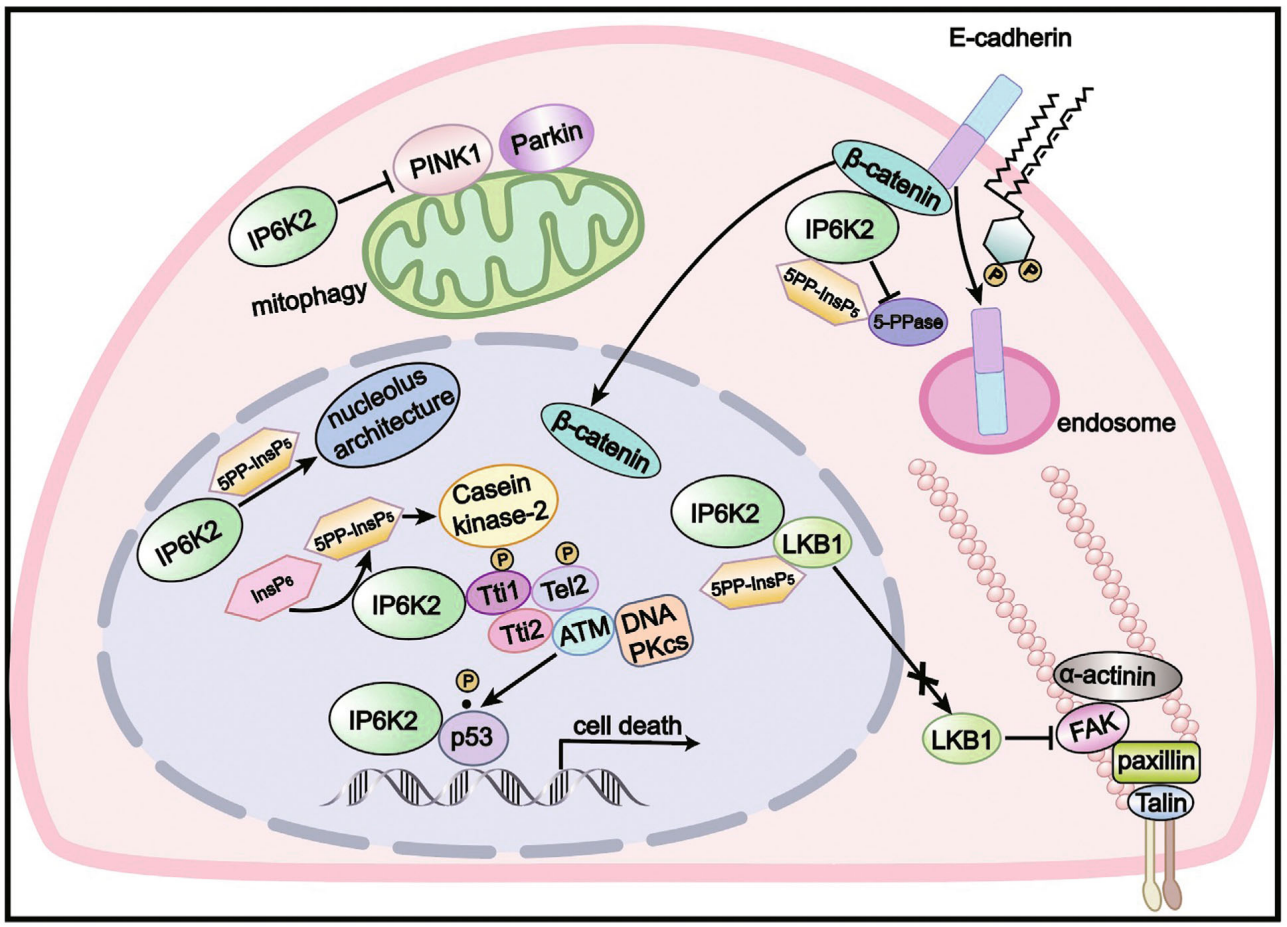
Mechanisms of action of IP6K2. In the nucleus, IP6K2 modulates nucleolar architecture and promotes p53‐mediated cell death. Additionally, IP6K2 binds to LKB1 and sequesters it within the nucleus, thereby preventing LKB1‐mediated inhibition of FAK signaling. In the cytosol, IP6K2 attenuates mitophagy mediated by PINK1. Additionally, IP6K2 can induce endocytosis of E‐cadherin through its product 5PP‐InsP_5_, thereby promoting the nuclear translocation of β‐catenin.

IP6K2 is primarily located within the nucleus, and *via* its product 5PP‐InsP_5_, modulates nucleolar architecture [58]. In mouse fibroblast cells, IP6K2 produces up to 40% of intracellular 5PP‐InsP_5_ [59, 60]. A prominent role of IP6K2 is the regulation of cell apoptosis [61, 62]. Thus, increased expression of IP6K2 enhances the sensitivity of certain ovarian carcinomas to radiation and IFN‐β [63]. IP6K2 binds directly to p53, and this binding promotes p53‐mediated apoptosis while suppressing cell‐cycle arrest [64]. This effect requires 5PP‐InsP_5_, the product of IP6K2. 5PP‐InsP_5_ binds casein kinase‐2 to enhance phosphorylation of the TTT complex, thereby stabilizing DNA‐PKcs and ATM, leading to p53 phosphorylation at serine 15, which activates the cell death program in human cancer cells and in murine B cells [60]. Additionally, IP6K2 plays a role in mitophagy in a kinase‐independent manner [65] (Fig. 3).

Mice with an *Ip6k2* null mutation display grossly normal embryogenesis, development, fertility, and lifespan in laboratory animal facilities [59]. However, deletion of IP6K2 causes defects in synaptic influences of granule cells upon Purkinje cells as well as impairment of locomotor function [12]. Cerebella of IP6K2‐deleted mice produce less phosphocreatine and ATP and generate higher levels of reactive oxygen species and protein oxidative damage [66]. IP6K2 inhibition significantly alters expression levels of gene sets associated with mature neurons, neural progenitor/stem cells, and glial cells, as well as certain genes modulating neuronal differentiation and function. This implies critical roles of IP6K2 in the developmental and functional regulation of the enteric nervous system [67] (Table 2).

**Table 2:** The physiological functions of IP6K2.

	Mechanisms of action
Cellular functions	
1. IP6K2 mediates cell death	IP6K2 *via* its product 5PP‐InsP_5_ promotes p53‐mediated apoptosis
2. IP6K2 promotes cell migration	IP6K2 binds to LKB1 and sequesters it in the nucleus, thereby preventing LKB1‐mediated inhibition of FAK
3. IP6K2 attenuates mitophagy	IP6K2 suppresses PINK1‐mediated mitophagy pathway in a kinase‐independent manner
4. IP6K2 modulates nucleolar architecture	IP6K2 generates 5PP‐InsP_5_ to cause outer nucleolar granular region expansion
5. IP6K2 regulates cellular energy dynamics	IP6K2 binds to creatine kinase‐B, and promotes the generation of phosphocreatine
6. IP6K2 weakens the intestinal epithelial barrier	IP6K2, *via* its product 5PP‐InsP_5_, inhibits inositol 5‐phosphatases, thereby enabling PI(4,5)P_2_‐facilitated E‐cadherin endocytosis
*In vivo* functions	
1. IP6K2 regulates motor coordination	IP6K2 promotes parallel fiber‐Purkinje cell synapse formation
2. IP6K2 promotes enteric nervous system development	IP6K2 promotes gene expression, modulating neuronal differentiation and function

### Physiological functions and mechanisms of action of IP6K3

**Fig. 4:**
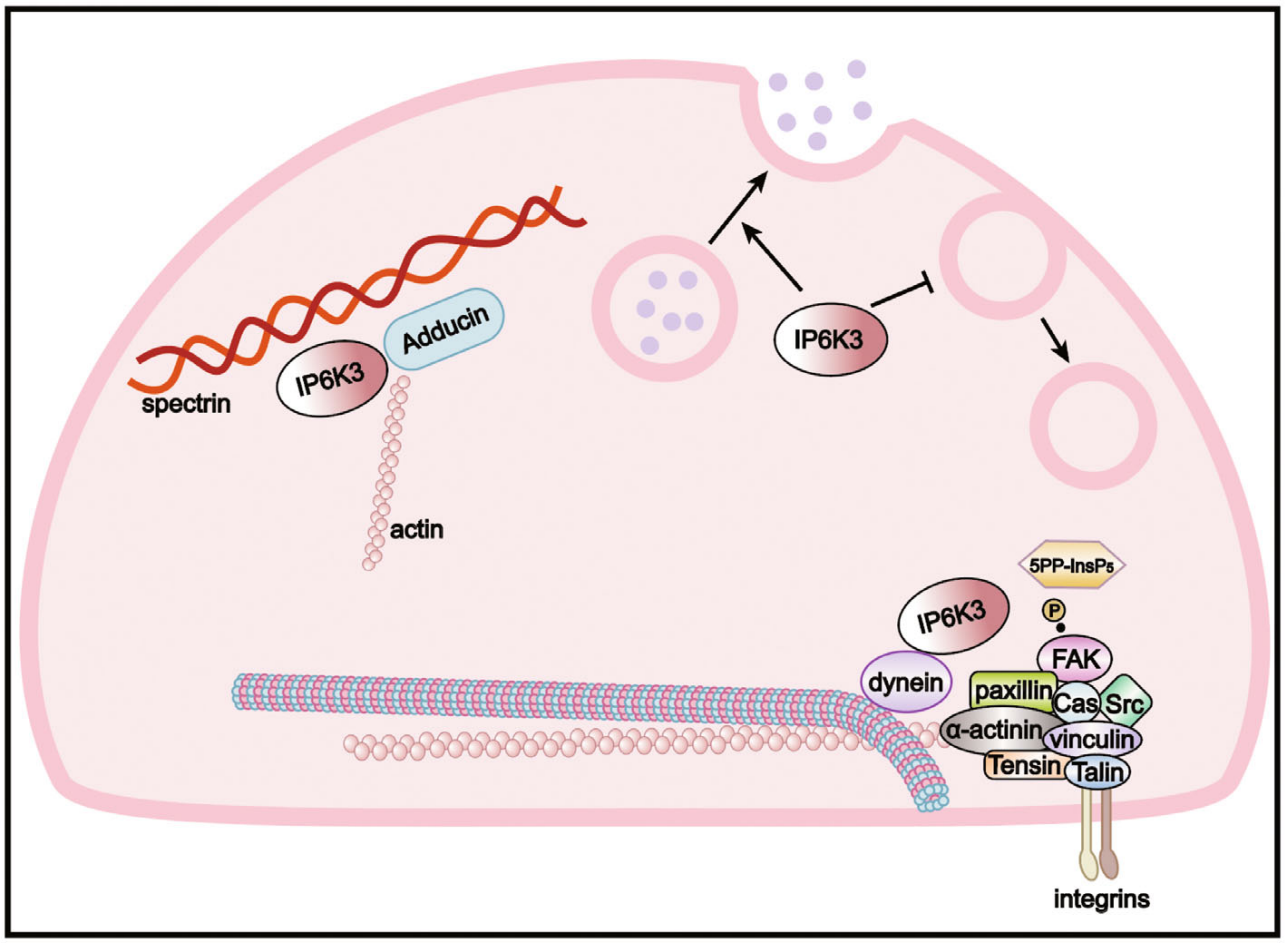
Mechanisms of action of IP6K3. At the cell membrane, IP6K3 collaborates with dynein and promotes focal adhesion turnover through its product, 5PP‐InsP_5_. IP6K3 also binds to spectrin and adducin, facilitating cytoskeleton organization. Additionally, IP6K3 enhances synaptic vesicle release while inhibiting synaptic vesicle recycling.

IP6K3 is highly expressed in the nervous system and skeletal muscle. In neuronal cells, IP6K3 coordinates with dynein intermediate chain 2 in a confined intracellular microenvironment to promote focal adhesion turnover [9] (Fig. 4). IP6K3 also regulates synaptic vesicle recycling [50]. In cerebellar Purkinje cells, IP6K3 physiologically binds to the cytoskeletal proteins adducin and spectrin to facilitate their functions [11]. Deletion of IP6K3 causes deficits in motor learning and coordination [11] and brain malformations [9]. Animal studies show that IP6K3 in skeletal muscle regulates metabolism and lifespan [68] (Table 3). In humans, IP6K3 might play a role in power performance [69].

**Table 3:** Physiological functions of IP6K3.

Functions	Mechanisms of action
1. IP6K3 regulates motor learning and coordination	IP6K3 binds adducin and spectrin to facilitate synapse formation in cerebellar Purkinje neurons
2. IP6K3 promotes neuronal development	In neurons, IP6K3 coordinates with dynein to localize to the cell membrane. Through its product 5PP‐InsP_5_, IP6K3 promotes focal adhesion turnover, thereby enhancing neuronal migration
3. IP6K3 promotes synaptic facilitation	IP6K3 promotes exocytosis of synaptic vesicle, but inhibits synaptic vesicle recycling
4. IP6K3 regulates energy expenditure in skeletal muscle	IP6K3 inhibits glucose metabolism

## Physiological functions and mechanisms of action of PPIP5Ks

Mammalian cells express two PPIP5Ks, namely PPIP5K1 and PPIP5K2. These enzymes can phosphorylate InsP_6_ and 5PP‐InsP_5_ to generate 1PP‐InsP_5_ and InsP_8_, respectively [70]. The concentration of cellular InsP_8_ is 5 to 10 times higher than that of 1PP‐InsP_5_, suggesting that InsP_8_ is the major product. PPIP5Ks also possess a phosphatase domain that can hydrolyze the 1‐β‐phosphate from either 1PP‐InsP_5_ or InsP_8_ to generate InsP_6_ and 5PP‐InsP_5_. The phosphatase activities of PPIP5Ks are strongly inhibited by phosphate [71]. Interestingly, when acting as a phosphatase, PPIP5Ks preferentially dephosphorylate InsP_8_ over 1PP‐InsP_5_, resulting in InsP_8_ being dephosphorylated about 10‐fold faster than 1PP‐InsP_5_. Recently, the crystal structures of the apo and substrate‐bound PPIP5K phosphatase domain from *S. cerevisiae* have been solved, confirming that the enzyme has a strong preference for InsP_8_ and is inhibited by inorganic phosphate [72]. Thus, the major enzymatic activities of PPIP5Ks are to phosphorylate 5PP‐InsP_5_ to InsP_8_ and to convert InsP_8_ back to 5PP‐InsP_5_.

PPIP5Ks create a ‘futile’ cycle between 5PP‐InsP_5_ and InsP_8_, as well as between InsP_6_ and 1PP‐InsP_5_. Since the 5PP‐InsP_5_/InsP_8_ pathway is the major metabolic route, it is plausible to assume that PPIP5Ks function within similar subcellular compartments as IP6Ks to modulate the local concentrations of 5PP‐InsP_5_ and InsP_8_ in a timely and precise manner. This may allow them to switch signals back and forth. For example, PPIP5Ks convert 5PP‐InsP_5_ to InsP_8_, relieving 5PP‐InsP_5_‐dependent Akt inhibition [73, 74]. Deleting PPIP5Ks elevates cellular 5PP‐InsP_5_ levels by two‐ to threefold [24], resulting in the upregulation of NUDT3 mRNA substrates and an increase in processing bodies [75].

### Physiological functions and mechanisms of action of PPIP5K1

The kinase activity of PPIP5K1 requires 5PP‐InsP_5_ as a substrate, indicating that PPIP5K1 kinase activity is influenced by IP6Ks. For example, PPIP5K1 promotes the activity of the phosphate exporter xenotropic and polytropic retrovirus receptor 1 (XPR1) *via* its product InsP_8_ [76]. Deletion of IP6K1/IP6K2 depletes 5PP‐InsP_5_, which subsequently depletes InsP_8_. This leads to a reduction in XPR1‐mediated phosphate efflux [77]. This phenotype is confirmed by an animal study that showed inhibiting IP6K activity leads to suppression of XPR1, thereby inhibiting cellular phosphate export and decreasing plasma phosphate levels [78]. Although both 5PP‐InsP_5_ and InsP_8_ can bind to XPR1, it is InsP_8_, the product of PPIP5K1, that promotes the activity of XPR1‐dependent phosphate efflux [76].

In addition to regulating phosphate efflux, PPIP5K1 also regulates phosphate uptake *via* its product InsP_8_, thereby affecting the stability of the PiT1 protein. Deleting PPIP5K1 downregulates PiT1 and thus reduces phosphate uptake [79]. This regulation of phosphate homeostasis by PPIP5K1 determines osteoblastic cell biomineralization [76].

**Fig. 5:**
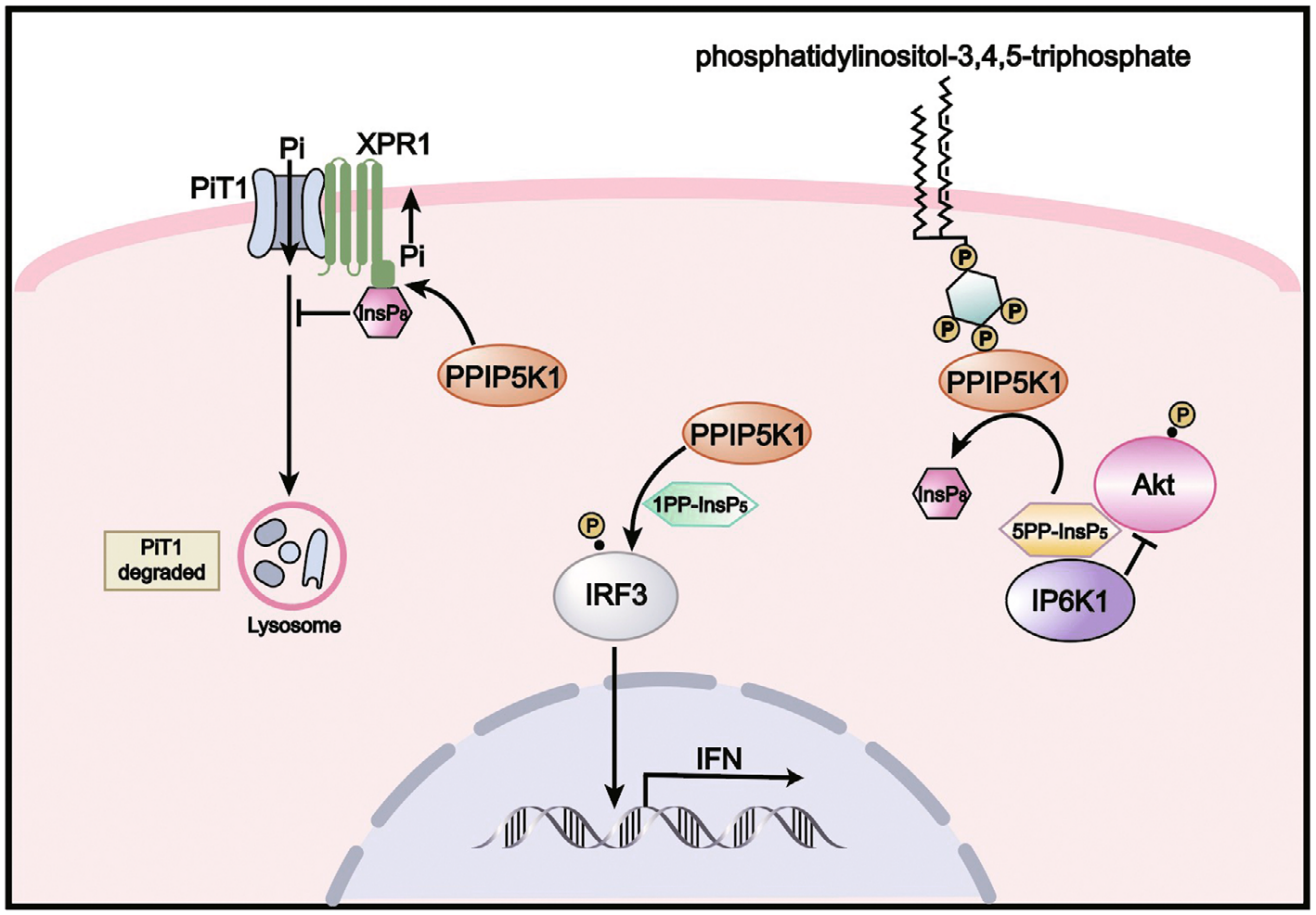
Mechanisms of action of PPIP5K1. PPIP5K1 is recruited to the cell membrane by phosphatidylinositol‐3,4,5‐triphosphate, where it converts 5PP‐InsP_5_ to InsP_8_, thereby relieving the inhibitory effect of 5PP‐InsP_5_ on Akt. PPIP5K1 regulates intracellular phosphate levels by activating XPR1‐dependent phosphate efflux through its product InsP_8_. Additionally, PPIP5K1 stabilizes PiT1 to regulate PiT1‐mediated phosphate uptake. Furthermore, PPIP5K1 activates the transcription factor IRF3 *via* its product 1PP‐InsP_5_.

The polyphosphoinositide‐binding domain in PPIP5K1 binds to phosphatidylinositol‐3,4,5‐triphosphate and is responsible for recruiting PPIP5K1 to the cell membrane. There, PPIP5K1 converts 5PP‐InsP_5_ to InsP_8_, relieving 5PP‐InsP_5_‐dependent inhibition of Akt [73]. Both phosphatidylinositol‐4,5‐bisphosphate and phosphatidylinositol‐3,4,5‐triphosphate inhibit the phosphatase activity of PPIP5K1, thereby enhancing the conversion of 5PP‐InsP_5_ to InsP_8_ [80]. Thus, PPIP5K1 can also modulate the activity of IP6Ks (Fig. 5).

PPIP5K1 contains an unusually long and evolutionarily conserved intrinsically disordered region, which is responsible for its interaction with multiple proteins [81]. PPIP5K1 also plays a role in cell migration [82]. Deleting PPIP5K1 decreases the motility of HeLa cells in a wound‐healing assay [81]. PPIP5K1 promotes innate immune responses to viral infection by generating 1PP‐InsP_5_, which enhances the phosphorylation and activation of IRF3, a transcription factor for interferon [83].

PPIP5K1 might be essential for maintaining sensorineural hearing function and male fertility because genetic studies suggest that deletion of *PPIP5K1* causes autosomal recessive mild‐to‐moderate sensorineural hearing loss [33] and male infertility [84]. However, more evidence is required to support these conclusions (Table 4).

**Table 4:** The physiological functions of PPIP5K1.

Functions	Mechanisms of action
1. PPIP5K1 promotes phosphate export	IP_8_, a product of PPIP5K1, binds to the SPX domain of XPR1 to activate XPR1‐mediated inorganic phosphate efflux
2. PPIP5K1 enhances phosphate uptake	PPIP5K1, *via* its product IP_8_, promotes PiT1‐mediated phosphate uptake by preventing its degradation
3. PPIP5K1 promotes Akt activation	PPIP5K1 converts 5PP‐InsP_5_ into IP_8_, which relieves 5PP‐InsP_5_‐mediated Akt inhibition
4. PPIP5K1 promotes cell migration	PPIP5K1 interacts directly with exocyst component 1
5. PPIP5K1 promotes innate immune responses	PPIP5K1, *via* its product 1PP‐InsP_5_, activates IRF3, which initiates the transcription of interferon
6. PPIP5K1 is required for sensorineural hearing function	Not known
7. PPIP5K1 is required for male fertility	Not known

### Physiological functions and mechanisms of action of PPIP5K2

**Fig. 6:**
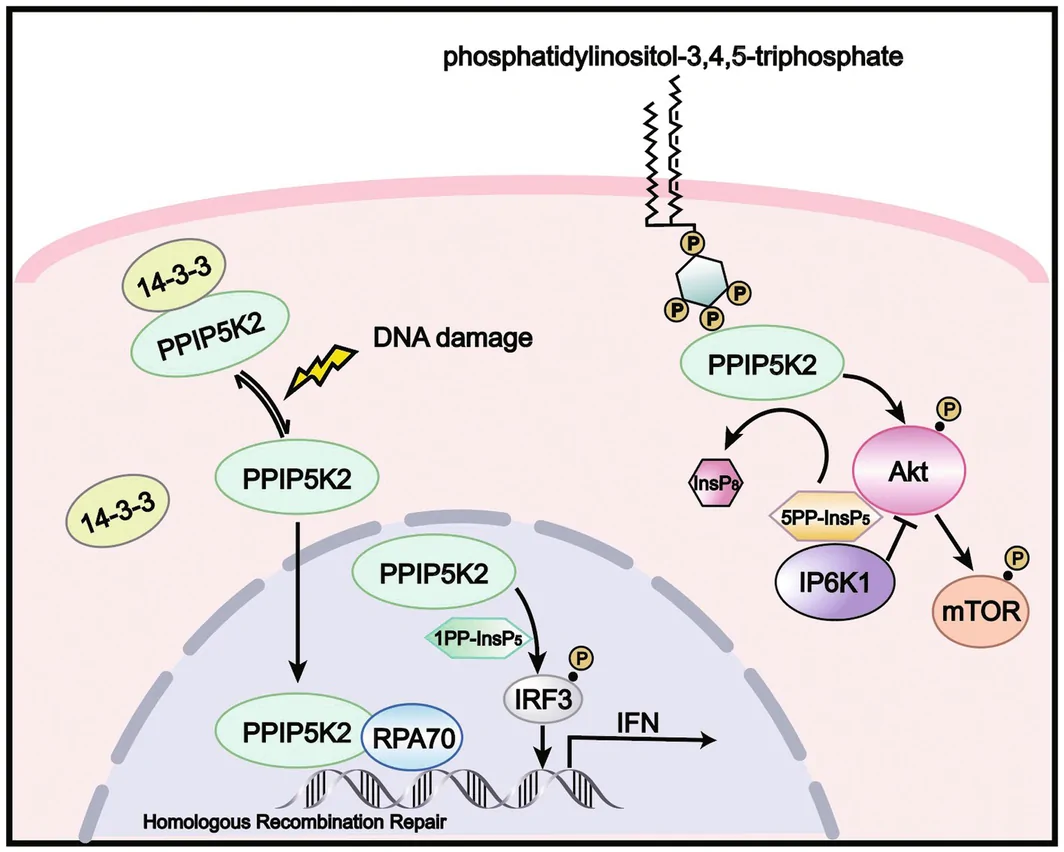
Mechanisms of action of PPIP5K2. PPIP5K2 relieves 5PP‐InsP_5_‐dependent inhibition of Akt by converting 5PP‐InsP_5_ to InsP_8_. In the nucleus, PPIP5K2 recruits RPA70 to the DNA damage site to promote homologous recombination repair. PPIP5K2 also activates IRF3 *via* its product 1PP‐InsP_5_ to induce interferon transcription.

PPIP5K2 promotes expansion and megakaryocyte/myeloid‐biased differentiation of hematopoietic stem cells by converting 5PP‐InsP_5_ to InsP_8_, which relieves 5PP‐InsP_5_‐dependent inhibition of Akt [74]. PPIP5K2 possesses a functional nuclear translocation sequence [85], allowing it to move to the nucleus. There, it acts as a scaffold protein to promote homologous recombination repair by binding and recruiting RPA70 to the DNA damage site [86]. PPIP5K2 can also promote innate immune responses to viral infection by producing 1PP‐InsP_5_, which activates IRF3‐dependent interferon transcription [83] (Fig. 6).

PPIP5K2 is required for cornea function. Whole exome and genome sequencing in familial keratoconus patients identifies mutations in PPIP5K2, leading to increased kinase activity and decreased phosphatase activity [34]. The role of PPIP5K2 in keratoconus pathogenesis has been validated in mouse models [34, 87]. In addition, the Arg837His mutation in PPIP5K2, which reduces its phosphatase activity and elevates its kinase activity, is associated with hearing loss in humans and mice [88] (Table 5).

**Table 5:** The physiological functions of PPIP5K2.

Functions	Mechanisms of action
1. PPIP5K2 promotes expansion and megakaryocyte/myeloid‐biased differentiation of hematopoietic stem cells	PPIP5K2 converts 5PP‐InsP_5_ to InsP_8_, which relieves 5PP‐InsP_5_‐dependent inhibition of Akt
2. PPIP5K2 promotes homologous recombination repair	PPIP5K2 binds and recruits RPA70 to the DNA damage site
3. PPIP5K2 promotes innate immune responses	PPIP5K2, *via* its product 1PP‐InsP5, activates IRF3, which initiates the transcription of interferon
4. PPIP5K2 is required for cornea function	Not known
5. PPIP5K2 is required for sensorineural hearing function	Not known

## IP6Ks and PPIP5Ks in diseases

A growing body of evidence suggests that IP6K1 plays a role in mediating age‐related diseases, and these pathological processes could be mitigated through pharmacological inhibition of IP6K1. IP6K2 has been recognized as a mediator of cell death, especially in the context of neurodegenerative diseases. IP6K3 has been implicated in the pathogenesis of late‐onset Alzheimer's disease as well as inflammatory conditions. Thus, IP6Ks may represent promising therapeutic targets for the management of age‐related diseases, such as metabolic disorders, cardiovascular diseases, and neurodegenerative diseases. Moreover, PPIP5K1 and PPIP5K2 have been demonstrated to contribute to cancer development, making them potential targets for cancer treatment interventions (Table 6).

**Table 6:** Therapeutic potential of targeting IP6Ks and PPIP5Ks.

Treatment target	Potential therapeutic applications	Beneficial effects
IP6K1	Metabolic dysfunction	Inhibition of IP6K1 enhances energy expenditure, lowers blood glucose, and ameliorates diet‐induced obesity and obesity‐induced bone loss
	Cardiovascular diseases	Inhibition of IP6K1 attenuates atherosclerosis, alleviates hyperglycemia‐induced endothelial dysfunction, and protects cardiomyocytes from ischemia–reperfusion injury
	Cancers	Debatable
IP6K2	Neurodegenerative diseases	Inhibition of IP6K2 might be able to attenuate neuronal death in Huntington's disease, amyotrophic lateral sclerosis, and Parkinson's disease
	Cancers	Debatable
IP6K3	Undetermined	Undetermined
PPIP5K1	Cancers	Inhibition of PPIP5K1 prevents cancer development
PPIP5K2	Cancers	Inhibition of PPIP5K2 prevents cancer development. Additionally, PPIP5K2 is linked to a worse prognosis

### IP6K1 mediates age‐related diseases beyond early development

Higher expression levels of IP6K1 are associated with an increased risk of developing age‐related diseases. For instance, IP6K1 is upregulated in aged and diabetic organs [17, 89] and is downregulated by high‐intensity exercise [30]. Disruption of the *IP6K1* gene has been found in a family with type 2 diabetes mellitus [29]. IP6K1 has been proposed to contribute to the dysregulation of nutrient uptake in human skeletal muscle [31] and to mediate age‐induced changes in bone marrow [90].

Genetic studies utilizing *Ip6k1* KO animals provide evidence for the involvement of IP6K1 in metabolic and cardiovascular diseases. Global deletion of IP6K1 enhances temperature‐modulated energy expenditure, which reduces carbohydrate‐ and fat‐induced weight gain [26]. Adipocyte‐specific deletion of IP6K1 reduces diet‐induced obesity by enhancing AMPK‐mediated thermogenesis [91]. Endothelial‐specific overexpression of IP6K1 exacerbates hyperglycemia‐induced vascular endothelial senescence, whereas endothelial‐specific deletion of IP6K1 alleviates this effect [17].

Pharmacological studies have demonstrated that inhibiting IP6K1 exerts beneficial effects in attenuating metabolic and cardiovascular diseases [92]. Blocking IP6K1 activity ameliorates diet‐induced obesity and insulin resistance [93]. Inhibiting IP6K1 confers atheroprotection by elevating circulating apolipoprotein A‐1 [18], and protects the heart against ischemia‐reperfusion injury by elevating plasma adiponectin [19]. Pharmacological inhibition of IP6K1 also protects mice against obesity‐induced bone loss [94].

IP6K1 has been implicated in cancer behavior. Animal studies have demonstrated that IP6K1 deletion can inhibit cancer cell invasion [95, 96]. Moreover, IP6K1 has been identified as a target for an anti‐angiogenic factor [39]. In contrast, IP6K1 deletion in mice was shown to accelerate the growth of xenografted tumors by disrupting the tumor‐immune microenvironment [97]. Thus, the precise roles of IP6K1 in cancers remain enigmatic. Notably, IP6K1 deletion does not induce spontaneous tumor formation in animal models, despite activating Akt signaling [55].

### IP6K2 participates in neurodegenerative diseases and tumors

IP6K2 is primarily a nuclear protein. Its translocation from the nucleus to the cytoplasm can cause downregulation of Akt signaling, which contributes to cell death in Huntington's disease [98]. Similarly, in a mouse model of amyotrophic lateral sclerosis, the expression levels of IP6K2 are increased, and it is translocated from the nucleus to the cytoplasm [99]. A human genetic study suggests that IP6K2 might also play a role in cognitive dysfunction in Parkinson's disease [100]. This IP6K2‐mediated cell death effect is mediated by its product, 5PP‐InsP_5_. Direct microinjection of 5PP‐InsP_5_ induces cell death [59]. Casein kinase‐2 phosphorylates IP6K2 to enhance its ubiquitination and subsequent degradation, leading to increased cell survival [101].

While elevated levels of IP6K2 are associated with a higher risk of neurodegenerative diseases, its role in cancer is more complex. On one hand, IP6K2 deletion in animals increases susceptibility to aerodigestive tract carcinoma [59]. Tumors that lack IP6K2 show resistance to HSV‐1 oncolysis, leading to poorer therapeutic outcomes [102]. Conversely, IP6K2 deletion can inhibit tumor growth and metastasis [23]. In glioma tissues and cell lines, IP6K2 is upregulated, and its overexpression in glioma cells enhances cell proliferation, migration, and invasion [103]. A recent study indicates that IP6K2 may serve as a favorable prognostic factor in breast cancer. Specifically, improved prognosis was observed in patients with high IP6K2 gene expression compared to those with low IP6K2 gene expression among systemically untreated patients from Swedish low‐risk and Dutch cohorts [32]. In contrast, the upregulation of IP6K2 in human colorectal cancers is associated with worse disease‐free survival. IP6K2, through its product 5PP‐InsP_5_, promotes the endocytosis of E‐cadherin and enhances the transcriptional activities of β‐catenin [104].

### IP6K3 might play a role in neurodegenerative and inflammatory diseases

Single‐nucleotide polymorphism analysis suggests that IP6K3 might play a role in late‐onset Alzheimer's disease [105], inflammatory bowel disease [106], Hashimoto's thyroiditis [107], and longevity [108]. However, the underlying mechanisms and potential applications of IP6K3 as a druggable target remain to be fully elucidated.

### PPIP5K1 and PPIP5K2 promote cancer development

Overexpression of PPIP5K1 decreases p53 activity and thus prevents cell death induced by cytotoxic agents [109]. In contrast, deletion of PPIP5Ks in HCT116 colon cancer cells upregulates p53 and p21 and inhibits proliferation, indicating that PPIP5Ks are potential targets for tumor therapy [24]. Similarly, PPIP5K2 promotes cancer proliferation and metastasis by activating the Akt/mTOR pathway [110]. PPIP5K2 protein levels can be regulated by the ubiquitin‐proteasome pathway [111]. Higher expression levels of PPIP5K2 are associated with worse prognosis in patients with colorectal carcinoma [86]. This evidence indicates that PPIP5Ks might be potential targets for cancer treatment.

## Conclusions and perspectives

In this review, we illustrate the roles and mechanisms of action of IP6Ks and PPIP5Ks in physiology and diseases. IP6Ks catalyze the phosphorylation of InsP_6_ to produce 5PP‐InsP_5_, the most abundant inositol pyrophosphate in mammalian cells. PPIP5Ks primarily function downstream of IP6Ks and establish a ‘futile’ cycle between 5PP‐InsP_5_ and InsP_8_. They phosphorylate 5PP‐InsP_5_ to generate InsP_8_, which can then be converted back to 5PP‐InsP_5_. Thus, we propose that IP6Ks serve as a gate to initiate inositol pyrophosphate signaling, while PPIP5Ks act as a switch by interconverting 5PP‐InsP_5_ and InsP_8_. For instance, inhibiting IP6K activity effectively shuts down the activities of both 5PP‐InsP_5_ and InsP_8_. The conversion of 5PP‐InsP_5_ to InsP_8_ by PPIP5Ks alleviates the 5PP‐InsP_5_‐dependent inhibition of Akt and activates XPR1‐dependent phosphate efflux. The mechanisms of action of 5PP‐InsP_5_ and InsP_8_ can be directly demonstrated using chemically synthesized inositol pyrophosphates and their analogues [112, 113, 114, 115, 116, 117].

Animals with a null mutation in a single *Ip6k* or *Ppip5k* gene live a normal lifespan but display developmental defects. Whether 5PP‐InsP_5_ and InsP_8_ are essential for life remains to be determined, as there are no *Ip6k1*/*Ip6k2*/*Ip6k3* triple‐knockout animals or *Ppip5k1*/*Ppip5k2* double‐knockout animals available.

While IP6Ks and PPIP5Ks are crucial for early development, preclinical and clinical studies have shown that they also contribute to pathological processes beyond the young adult stage. For example, IP6K1 mediates metabolic disorders and cardiovascular diseases, IP6K2 and IP6K3 participate in neurodegenerative diseases, and PPIP5Ks promote cancer development. Inhibiting 5PP‐InsP_5_ biosynthesis with IP6K inhibitors has shown beneficial effects in attenuating diabetes and cardiovascular diseases in animal models. However, it is still too premature to consider IP6Ks and PPIP5Ks as viable druggable targets for disease treatment. This will require a comprehensive understanding of their functions and mechanisms of action.

Several IP6K inhibitors have been developed [118, 119, 120, 121, 122, 123, 124]. However, more potent and specific IP6K isoform inhibitors are needed for precision interventions, as IP6Ks perform nonredundant functions. So far, there are no PPIP5K inhibitors available. Several studies have explored the reaction mechanisms of PPIP5K2 [125, 126, 127]. The crystal structures of the kinase domain of human PPIP5K2 have been solved [128]. Recently, the structure of fission yeast Asp1, which is the ortholog of human kinase PPIP5K, has also been solved [129]. These efforts provide support for the development of PPIP5K inhibitors.

## Author contributions

ZJ, AFC and CF conceptualized. CF wrote the manuscript. CX drew the diagrams. CW completed the tables. CX, CW, YC, WC, ZJ, AFC and CF revised and edited the manuscript.
